# Perceived adverse maternal control during childhood and striatal volume in healthy older adults: Differences between APOE ε4 carriers and non‐carriers

**DOI:** 10.1002/alz.087216

**Published:** 2025-01-09

**Authors:** Delia A Gheorghe, Morgane Kuenzi, Sarah Bauermeister

**Affiliations:** ^1^ Transylvanian Institute of Neuroscience, Cluj‐Napoca, Cluj Romania; ^2^ University of Oxford, Oxford United Kingdom; ^3^ Dementias Platform UK ‐ University of Oxford, Oxford United Kingdom

## Abstract

**Background:**

Early adversity has been reported as a risk factor for dementia. Adverse maternal control (MC) during childhood is believed to impact neural developmental pathways. Here we studied the associations between adverse MC and the volume of the dorsal striatum in older adults given evidence from the childhood adversity literature of structural reductions and altered reward processing. The contributing roles of APOE ε4 status together with multiple other known risk factors in brain aging were analysed.

**Method:**

We analysed data from the MRC NSHD Insight 46 study on the Dementias Platform UK Data Portal (N = 394, 48% female, 70 years). MRI acquisition and APOE ε4 genotyping are described in published cohort papers. Bilateral volumes of the caudate nucleus and putamen were obtained using the FMRIB Software Library. MC was quantified using the Parental Bonding Instrument (PBI). Using k means clustering, participants were grouped in low, medium, and high MC groups. Analyses controlled for sex, age, socioeconomic status in late adulthood and childhood, speed of pubertal growth, birthweight, BMI and MMSE score at the time of brain scanning.

**Result:**

Multivariate tests were followed up with Bonferroni‐corrected comparisons. Smaller bilateral caudate nuclei and reduced wellbeing were found in the high MC group compared to the low MC group. MC effects on the brain were not mediated by wellbeing. When considering the role of APOE ε4, Roy’s largest root revealed a significant interaction between MC and APOE ε4 status. Corrected follow‐up comparisons showed that in non‐carriers only, high MC was associated with smaller left caudate, left putamen volumes as well as reduced wellbeing, compared to the low MC group. Separately, in a smaller sample of APOE ε2 carriers (N = 51), the left caudate volume was smaller in high MC group.

**Conclusion:**

Our study showed that regions implicated in reward processing are smaller in individuals who experienced adverse MC during development. These effects were present in APOE ε4 non‐carriers. APOE ε4 effects are detectable in the dorsal striatum in later life in preclinical populations. Whether genotype masks stress effects on the brain in APOE ε4 carriers is yet to be established.

